# Low Latency Estimation of Motor Intentions to Assist Reaching Movements along Multiple Sessions in Chronic Stroke Patients: A Feasibility Study

**DOI:** 10.3389/fnins.2017.00126

**Published:** 2017-03-17

**Authors:** Jaime Ibáñez, Esther Monge-Pereira, Francisco Molina-Rueda, J. I. Serrano, Maria D. del Castillo, Alicia Cuesta-Gómez, María Carratalá-Tejada, Roberto Cano-de-la-Cuerda, Isabel M. Alguacil-Diego, Juan C. Miangolarra-Page, Jose L. Pons

**Affiliations:** ^1^Neural Rehabilitation Group, Spanish National Research Council, Cajal InstituteMadrid, Spain; ^2^Sobell Department of Motor Neuroscience and Movement Disorders, Institute of Neurology, University College LondonLondon, UK; ^3^Motion Analysis, Ergonomics, Biomechanics and Motor Control Laboratory, Department of Physical Therapy, Occupational Therapy, Rehabilitation and Physical Medicine, Faculty of Health Sciences, Rey Juan Carlos UniversityMadrid, Spain; ^4^Neural and Cognitive Engineering Group, Centro de Automática y Robótica, Universidad Politécnica de Madrid (UPM), Spanish National Research CouncilMadrid, Spain

**Keywords:** electroencephalography, motor-related cortical potentials, event-related desynchronization, functional electrical stimulation, stroke, neurorehabilitation

## Abstract

**Background:** The association between motor-related cortical activity and peripheral stimulation with temporal precision has been proposed as a possible intervention to facilitate cortico-muscular pathways and thereby improve motor rehabilitation after stroke. Previous studies with patients have provided evidence of the possibility to implement brain-machine interface platforms able to decode motor intentions and use this information to trigger afferent stimulation and movement assistance. This study tests the use a low-latency movement intention detector to drive functional electrical stimulation assisting upper-limb reaching movements of patients with stroke.

**Methods:** An eight-sessions intervention on the paretic arm was tested on four chronic stroke patients along 1 month. Patients' intentions to initiate reaching movements were decoded from electroencephalographic signals and used to trigger functional electrical stimulation that in turn assisted patients to do the task. The analysis of the patients' ability to interact with the intervention platform, the assessment of changes in patients' clinical scales and of the system usability and the kinematic analysis of the reaching movements before and after the intervention period were carried to study the potential impact of the intervention.

**Results:** On average 66.3 ± 15.7% of trials (resting intervals followed by self-initiated movements) were correctly classified with the decoder of motor intentions. The average detection latency (with respect to the movement onsets estimated with gyroscopes) was 112 ± 278 ms. The Fügl-Meyer index upper extremity increased 11.5 ± 5.5 points with the intervention. The stroke impact scale also increased. In line with changes in clinical scales, kinematics of reaching movements showed a trend toward lower compensatory mechanisms. Patients' assessment of the therapy reflected their acceptance of the proposed intervention protocol.

**Conclusions:** According to results obtained here with a small sample of patients, Brain-Machine Interfaces providing low-latency support to upper-limb reaching movements in patients with stroke are a reliable and usable solution for motor rehabilitation interventions with potential functional benefits.

## Introduction

Upper-limb function recovery after a stroke is in many cases insufficient despite intensive physical therapy. In order to actually get meaningful functional changes in these patients, it has been suggested that traditional physical therapies need to be paralleled with brain modulation interventions aimed to guide plastic changes in the brain (Belda-Lois et al., 2011).

Experimental neuromodulation paradigms using electrophysiological acquisition and stimulation techniques to produce long-term plastic changes at supraspinal and spinal levels have been proposed to treat motor dysfunction in stroke (Lefaucheur, 2006; Daly and Wolpaw, 2008). Among these, paradigms using Brain-Machine Interfaces (BMI) linking cortical motor-related activity with afferent information from limbs have been used to efficiently induce cortical plastic changes in healthy subjects (Xu et al., 2014b; Kraus et al., 2015) and in patients (Ramos-Murguialday et al., 2013; Várkuti et al., 2013).

The electroencephalographic (EEG) activity over the premotor and motor cortical areas presents characteristic variations in the periods before self-initiated movements. Two main motor related EEG patterns are known to reflect mental states related to motor planning and execution processes: the Bereitschaftspotential (BP; Shibasaki and Hallett, 2006) and the Event-Related Desynchronization (ERD; Pfurtscheller and da Silva, 1999). ERD and BP have been used in BMI experiments aimed to improve motor neurorehabilitation (Bhagat et al., 2016; Grimm et al., 2016; Lopez-Larraz et al., 2016). Previous studies have used these cortical patterns to detect the onset of voluntary movements in healthy subjects with temporal precisions of 200–500 ms with respect to the onset of muscle activations in the limbs (Lew et al., 2014; Xu et al., 2014a). The possibility of identifying this information of motor intentions allows establishing a tight temporal association of movement-specific cortical activations with proprioceptive afferent feedback from the moved limbs for rehabilitation purposes. Based on this idea, previous studies conditioned the cortico-muscular descending tract to the lower-limbs in control subjects and stroke patients by temporally associating motor intentions to perform analytical ankle movements with electrical or mechanical stimuli (Xu et al., 2014b). These studies showed that significant plastic changes were visible after a single session intervention if small latencies between the cortical activations and the peripheral stimuli were maintained. Moreover, this intervention concept has proven to be potentially beneficial for stroke rehabilitation (Mrachacz-Kersting et al., 2016, 2017).

Mostly, studies of low-latency detectors of motor intentions with the upper limb have only been carried out in offline conditions. Moreover, no interventions so far have tested BMI platforms decoding pre-movement BP and ERD patterns online in patients with brain damage due to a stroke. While BP detections online in healthy subjects doing ankle dorsiflexions have demonstrated to be reliable for BMI approaches, BPs in upper-limb movements (Hadsund et al., 2016; Martínez-Expósito et al., 2017), and specially in stroke patients (Daly et al., 2006) present particularities that make them less reliable for BMI applications, which may limit their usability in BMIs. Recently, it was demonstrated that an appropriate combination of BP- and ERD-based classifiers could lead to reliable and low-latency estimation of stroke patients' upper-limb motor intentions (Ibáñez et al., 2014a,b). In addition, it was shown that the use of Functional Electrical Stimulation (FES) can assist patients to perform functional complex (multi-joint) movements (Resquin et al., 2016). Here it is hypothesized that, in patients with a stroke and chronic arm motor dysfunction, the possibility of timely matching motor intentions with FES assisting specific motor functions opens a window for targeted neuromodulation interventions aimed at improving function-specific motor neural circuits. The simultaneous neuromodulation of ERD and BP phenomena might induce changes in cortical activity related to both motor planning and execution, unlike the existent approaches, thus boosting neurorehabilitation. To achieve this, it needs to be assessed the feasibility and impact of EEG-based (ERD + BP) low-latency decoder of motor intentions triggering FES in upper-limbs in patients along a certain period of time.

In this study, the ability of a BMI system to assist upper-limb functional movements of stroke patients based on their pre-movement cortical changes decoded online and on a single-trial basis was tested for the first time. Moreover, the study reports results of a multisession intervention (eight sessions during 1 month) using FES to assist upper-limb reaching movements of four chronic stroke patients when EEG-based low-latency estimations of motor intentions are detected. The BMI system performance, patients' functional changes as well as their subjective reports regarding the received intervention are used to discuss on potential benefits of the proposed intervention.

## Materials and methods

### Patients

Four chronic stroke patients (age 54 ± 12 years, mean ± *SD*; all males) with a lesion in the territory of the middle cerebral artery and a predominance of brachial hemiparesis were recruited for this study (see details in Table 1). Patients met the following inclusion criteria: (i) ability to manipulate most objects; (ii) spasticity less than or equal to two in the Modified Ashworth Scale; (iii) ability to understand instructions and actively participate in tasks. Patients with cognitive decline, sensory aphasia, visual impairment, behavioral disorders, articular rigidity, irreversible contractures and dysmetria, and those who had been treated with botulinum toxin or baclofen <6 months before the start of the study were excluded from the study.

**Table 1:** **Patients' clinical data**.

**Pat. Code**	**Age**	**Gender**	**Stroke type**	**Upper limb affected**	**Years since stroke**	**Fügl-Meyer index**	**Stroke impact scale**	**Rh. sessions /week**
P1	54	Male	Ischem.	L	3	61	64	2
P2	54	Male	Hemorr.	R	4	83	66	2
P3	69	Male	Hemorr.	L	4	65	44	0
P4	40	Male	Hemorr.	L	5	81	73	2

The experimental protocol for this study was approved by the Ethical Committee of the “Universidad Rey Juan Carlos” (Alcorcón, Spain) and warranted to be in accordance with the Declaration of Helsinki. All patients signed a written informed consent.

### Study protocol

All experiments were carried out in a sound- and light-attenuated ward of a clinical university. During BMI interventions, patients were seated on comfortable seats and with their arms resting on a desk and movements were performed with the affected upper limbs of the patients.

**Figure 1:**
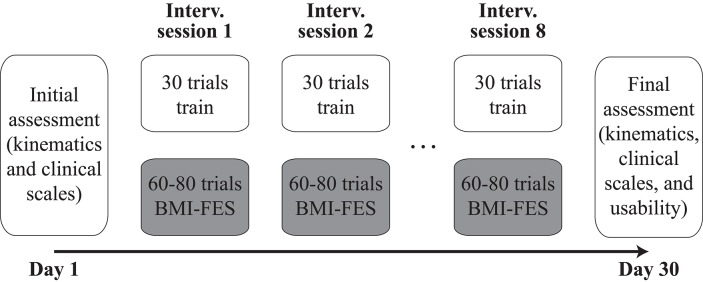
**Structure of the intervention carried out with each patient**.

Patients participated in 10 sessions carried out in different days during 1 month (see Figure 1). The whole intervention with each patient consisted of eight BMI-FES sessions (two sessions per week). Two additional sessions, right before and after the intervention phase, were scheduled to assess patients' functional evolution and their subjective evaluation of the received intervention (the latter only being carried out at the end of the whole process).

### Assessment sessions

#### Clinical scales

Clinical experts performed functional tests in the first and last sessions to analyse possible patients' improvements.

Upper-extremity sensorimotor function was assessed using Fügl-Meyer Assessment for Upper Extremities (FMA-UE). The four domains evaluated included: upper-extremity motor function (maximum score = 66), sensory function (maximum score = 12), passive joint motion (maximum score = 24), and joint pain (maximum score = 24). Items were scored on a 3-point ordinal scale from 0 (cannot perform) to 2 (performs fully). Summative scores were generated for each domain, scores ranged between 0 and 126 (Duncan et al., 1992; Wagner et al., 2008).

The Stroke Impact Scale 16 (SIS-16) was used to assess patients' health status following stroke. Duncan et al. (2003) developed the SIS-16 to assess physical function in patients with stroke using items from the composite physical domain of the Stroke Impact Scale (SIS) version 3.0. The SIS-16 can differentiate lower levels of disability. The SIS-16 consists of 16 items: seven activities of daily living items, eight mobility items, and a single hand function item. Each item is rated in a 5-point Likert scale in terms of the difficulty the patient has experienced in completing each item. Summative scores are generated for each domain. Scores range from 16 to 80 (Duncan et al., 2003).

#### Kinematics analysis

To analyse kinematics, patients performed five repetitions of the reaching task while being measured with a motion capture platform based on the optoelectronic system Vicon Motion (Oxford Metrics, Oxford, UK). Patients sat on a comfortable chair close to a desk. The patient-to-desk distance was 8-10 cm and the angle of the chair was 90°–100°. In the starting position the patient's trunk rested firmly against the back of the chair. Patients were asked to put their hands on the desk (palms down) with shoulder at around 20° of abduction and elbow at around 95° of flexion. A hard plastic glass (diameter = 5.5 cm, height = 15 cm) was used as target. The glass was placed on the desk in line with the patient's sternum and at a distance equal to 75% of the maximum reachable distance with the paretic arm.

Patients were instructed to reach the glass from the starting position using their paretic hand. All patients practiced the reaching task before motion capture trials. Once this phase was completed, a static calibration recording was performed. Using this recording, it was checked that each marker was visible from the scanning cameras and analyzed movements were registered. In these, after the verbal instruction “Get ready…go,” patients had to lift the arm and reach and grasp the glass at a comfortable speed (similar to the one used in the BMI-FES interventions). Three seconds after reaching the target patients had to move back to the initial position. The time needed to perform the movement was defined as the time interval between the hand movement onset until the hand reached the glass. We analyzed the shoulder, elbow and thorax positions when the hand reached the glass.

#### Satisfaction assessment

We evaluated patients' perceived comfort and acceptability of the BMI-FES platform. Five items are rated on a Likert-type scale from 1 to 5 (strongly disagree—strongly agree): (1) “you are satisfied with the intervention”; (2) “this intervention has been useful in order to carry out activities of daily living”; (3) “you would recommend this intervention to other subject in the same situation”; (4) “The instrumentation is uncomfortable.” The arithmetic mean across all items provides the total satisfaction score.

### Intervention sessions

#### BMI-FES platform

During the intervention sessions movements of the paretic arm were measured with solid-state gyroscopes, which allowed easy and robust recordings of transitions between resting and movement phases (Ibáñez et al., 2014a). Two gyroscopes (Technaid S.L., Madrid, Spain) were placed on the distal third of the forearm, and the middle of the arm. Data were sampled at 100 Hz and stored in a PC running a real-time OS (QNX Software Systems, Ottawa, Canada).

EEG signals were recorded from 31 positions (AFz, F3, F1, Fz, F2, F4, FC3, FC1, FCz, FC2, FC4, C5, C3, C1, Cz, C2, C4, C6, CP3, CP1, CPz, CP2, CP4, P3, P1, Pz, P2, P4, PO3, PO4, and Oz, all according to the International 10–20 system) with active Ag/AgCl electrodes (Acticap, Brain Products GmbH, Germany). The reference was set to the voltage of the earlobe contralateral to the arm moved. AFz was used as ground. The signal was amplified (gUSBamp, g.Tec GmbH, Austria) and sampled at 256 Hz. A standard PC was used to acquire and process the EEG data using a custom-made Simulink model (The Mathworks Inc., Natick MA, USA). This PC sent digital signals to the real-time PC using a USB DAQ (USB-6008, National Instruments, Austin TX, USA).

FES was delivered at the anterior deltoids, triceps and wrist extensors with a multichannel monopolar neurostimulator with charge compensated pulses (UNA Systems, Belgrade, Serbia). Traditional surface electrodes (Pals Platinum—rectangle 5 × 5 cm) were used. The common electrode was located on the oleocranon. Pulse width and frequency were set to 350 μs and 30 Hz, respectively. FES current was adapted in each session with each patient to achieve comfortable stimulation levels that elicited muscle contractions. Current values ranged between 20 and 50 mA (depending on the motor threshold of each muscle and to the patients' acceptance of the received stimulation). Due to the FES configuration and to the weight of the patients' arms, FES alone was not able to lift the arm unless it was successfully triggered by the BMI when patients attempted to perform the reaching task (in which case it provided assistance to the attempted movement). The stimulator was controlled by the PC storing gyroscopic data, which in turn received activation commands from the computer recording the EEG activity via a digital signal. Each time FES was activated it was done in a sequential manner (first deltoids and 250 ms later triceps and wrist extensors) so that the arm could first be lifted from the table and then extended toward the target.

#### EEG-based detection of the motor intentions with low latencies

**Figure 2:**
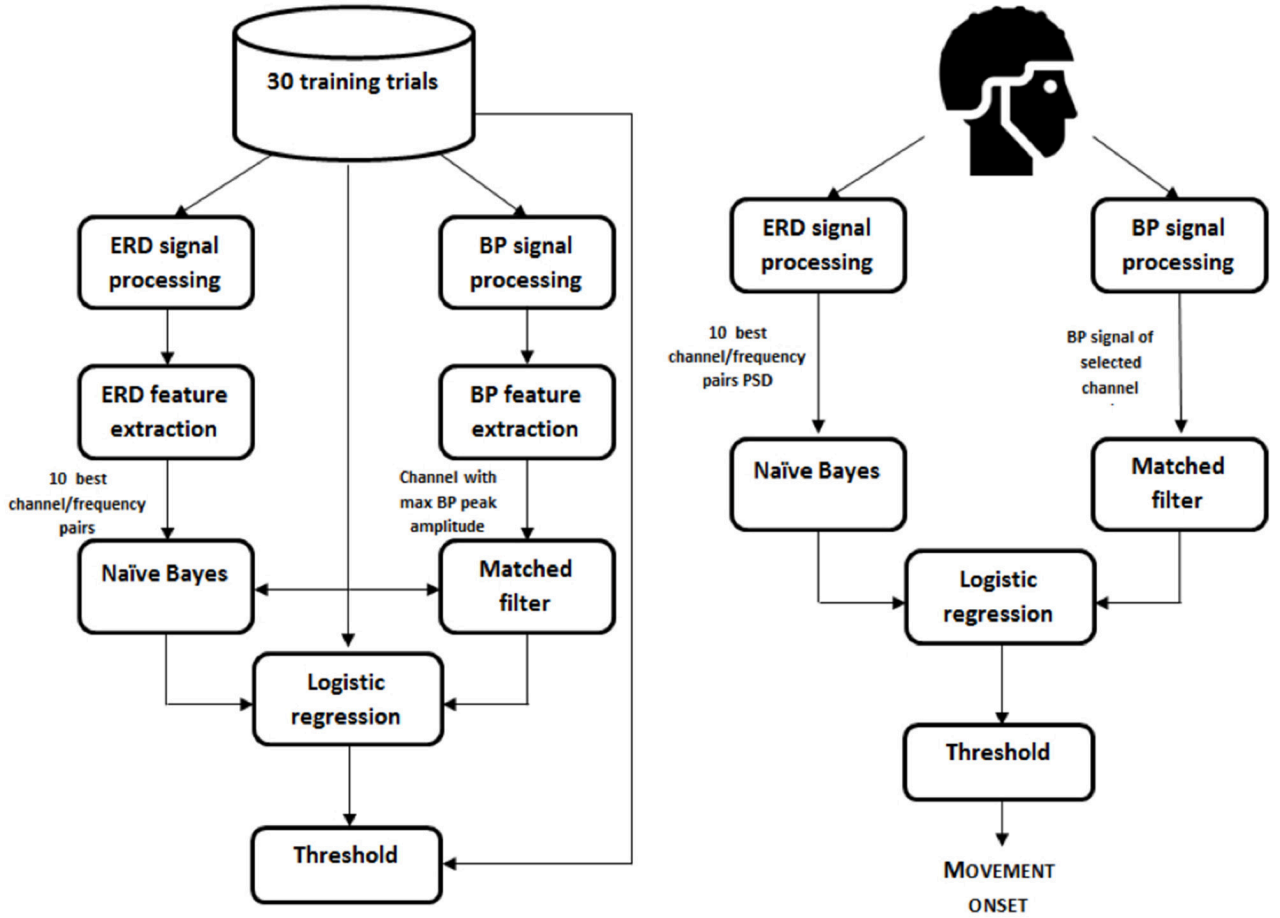
**Movement onset decoder scheme**. Left, calibration; Right, online decoding.

The classifier used to detect movement intentions from EEG was based on the one presented in Ibáñez et al. (2014a). A logistic regression was used to detect the onset of the voluntary movements based on the characterization of the ERD and BP cortical patterns observed in patients (Figure 2).

##### Detection of the onsets of movements from gyroscopes

Locations of onsets of voluntary movements were estimated based on the gyroscopic signals. For this, gyroscopic recordings were low-pass filtered (Butterworth, order 3, <10 Hz). For each patient, the sensor that first showed changes during the execution of reaching movements (between the one placed on the forearm and the one on the arm) was used. The peak amplitude performing the movements was estimated in each session. A threshold amplitude for the detection of the onsets of the movements was set to 5% of this peak amplitude. Finally, visual correction of the detected onsets was carried out to ensure that involuntary and residual movements were not taken into account for training and BMI validation purposes.

##### Feature extraction

EEG signals recorded in the pre-intervention calibration trials in each session were used to extract the best features for the posterior online decoder.

For the ERD detection, band-pass filtering (Butterworth, 3th order, 6 Hz < f1, 35 > f2) and small Laplacian filtering were applied to the EEG signal. Power values in segments of 1.5 s and in the frequency range 7–30 Hz (with steps of 1 Hz) were obtained from the frontal, fronto-central, central, centro-parietal, and parietal channels. The Welch's method was used to get power estimations (Hamming windows of 1 s; 50% overlap). The estimations in the training trials from –3 to –0.5 s (with respect to the movement onsets) were labeled as examples of the resting state. Estimations at the movement onsets were labeled as examples of the movement state. The Bhattacharyya distance was used to select the 10 best channel-frequency pairs to build the classifier, i.e., the 10 with the largest distance between the resting state and the movement onset estimations.

For BP, a Butterworth low-pass filter (1 Hz > fc, 1st order) was applied to extract the low-frequency components of EEG signals. A modified version of the large laplacian filter using as reference the average information from eight peripheral channels in the EEG electrode layout was used in order to minimize the weight of individual reference channels (Ibáñez et al., 2014a). Three virtual channels were generated by subtracting the average recordings of channels F3, Fz, F4, C3, C4, P3, Pz, and P4 to channels C1, Cz, and C2. These three de-referenced central channels were considered since the late part of the BP typically presents a lateralization in upper-limb movements (Shibasaki et al., 1980) and the spatial distribution of motor cortical activations in stroke patients may be altered due to their brain lesions (Serrien et al., 2004). The average BP of the resulting channels was obtained using the training data. The channel showing the highest peak at the movement onset relative to the average amplitude in the interval [−3,−2] s (with respect to the movement onset) was selected for BP-based detection of movement onsets.

##### Classifier construction

A naïve Bayes classifier of independent features was used to detect the ERD pattern preceding the onset of the reaching movements by using 10 channel-frequency pairs previously selected.

A matched filter of length 1.5 s was designed using the selected channel for BP detection. The filter was obtained by removing the baseline level (first 500 ms) of all 1.5 s trials in the training dataset and then averaging the BPs.

To train the logistic regression classifier that combined ERD- and BP-based estimations of motor intentions, training examples of the resting condition were taken from outputs of both classifiers (ERD and BP) between −3 s and −0.5 s, and estimations between ± 125 ms with respect to the onsets of movements were used to model the movement state.

##### Online decoding

In the online decoding phase, the logistic regression classifier generated estimations of movement intentions every 100 ms. The decoder yielded a binary output depending on whether the probabilistic output from the logistic regression classifier was over or under a certain threshold, which in turn activated the FES. The threshold was obtained from the training dataset and, if needed, it was further adjusted based on the reports of the patient in a few number of pre-intervention calibration trials. The threshold was initialized following the criterion of maximizing the percentage of good trials (GT), i.e., trials with a true positive (TP), and with no false positives (FP). TP were movements detected by the BMI with a detection latency within the range of ±750 ms with respect to the reference onsets estimated with the gyroscopes. EEG-based movement intention detections during resting phases were considered FPs. The precision of the detector was characterized by computing the number of FP per minute (FP/min). The percentage of GT was obtained by counting the amount of trials with no FP and a TP. Finally, latencies of the TP with respect to the onsets of movements were computed to analyse the temporal accuracy of the system. The definition of all these metrics is further elaborated in Ibáñez et al. (2014a).

To achieve a stable BMI system, outputs of the BMI were processed by a block ensuring that consecutive FES stimuli were separated by at least 5 s of time.

#### BMI-FES intervention and fes configuration

Once the BMI system had been calibrated, the intervention phase of the session begun. Patients performed 60–80 movements assisted with FES triggered by the BMI. The specific amount of trials performed in each session depended on the patients' arousal and their willingness to continue. Patients were allowed to rest and talk in the middle of the sessions if they needed to. Throughout the intervention trials, patients were asked to concentrate and have the FES activated with their movements.

Each time a trigger from the BMI was received by the PC controlling the FES system, the stimulation pattern described in Section BMI-FES Platform was triggered.

#### Validation of the BMI performance

In order to validate the performance of the EEG-based decoder of motor intentions during the interventions, the GT and the detection latencies during the intervention trials were computed. To facilitate the evaluation of the BMI system, patients were instructed not to perform movements when FES stimulation arrived before they have planned to start the movement. The times at which FES stimuli were triggered were compared to the onsets of movements according to the data from gyroscopes.

### Statistical procedures

All statistical analyses were performed using SPSS 17.0 (IBM Corp., New York USA) and Matlab2011 (The Mathworks Inc., Natick MA, USA). Due to the small sample size, Shapiro-Wilk test was applied to check normality of BMI performance and clinical scores. Given that all analyzed samples violated the statistical normality, Wilcoxon signed-rank test was used to compare the clinical scales scores before and after the intervention. The Friedman test for repeated measures was used to compare the BMI performance scores between sessions. Only the sessions with data from all patients were included in the analysis. A linear least square fitting was applied to estimate the tendency of the BMI performance measures along sessions, obtaining the squared error *R*^2^ and the gradient of the line *m*.

All results are reported as the mean ± *SD*, and considered significant if *P* < 0.05.

## Results

### Patients' ERD and BP

**Figure 3:**
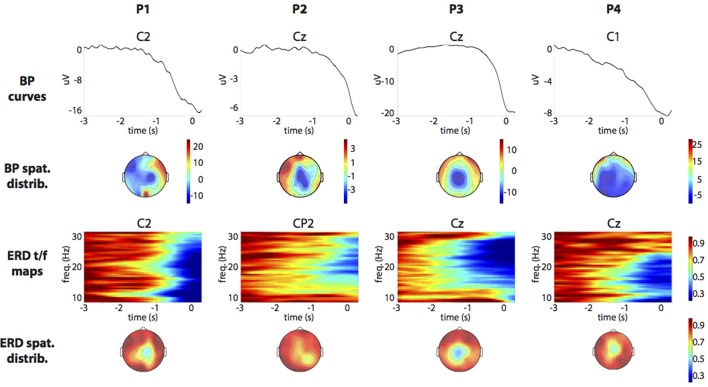
**Patients' ERD (8–30 Hz) and BP (0.05–1 Hz) patterns**. To optimize visualization, baseline was defined within [−3,−2] s and [−5,−3] s for BP and ERD, respectively. Average referencing was used for BP. Small Laplacian filters were used for ERD. BP and ERD of the most reactive EEG channels are shown in rows 1 and 3.

ERD and BP patterns were used by the BMI platform to control FES assistance. Figure 3 shows the average (across sessions) ERD and BP patterns of each patient taking part in the experiments. Fieldtrip's *ft_multiplotER* and *ft_multiplotTFR* functions were used to obtain the patterns (Oostenveld et al., 2011). As shown in the figure, there is a large variability between patients in terms of the magnitude of the ERD and BP patterns and in terms of their spatial, frequency, and temporal distributions. In all cases, ERD and BP start before the onset of the movements. In addition, it is observed that in all cases, the BP minimum peak is delayed several hundreds of milliseconds with respect to the movement onset. Neither the laterality nor the degree of change of the ERD/BP patterns showed a correlation with the patients' upper limb function.

### BMI performance

**Figure 4:**
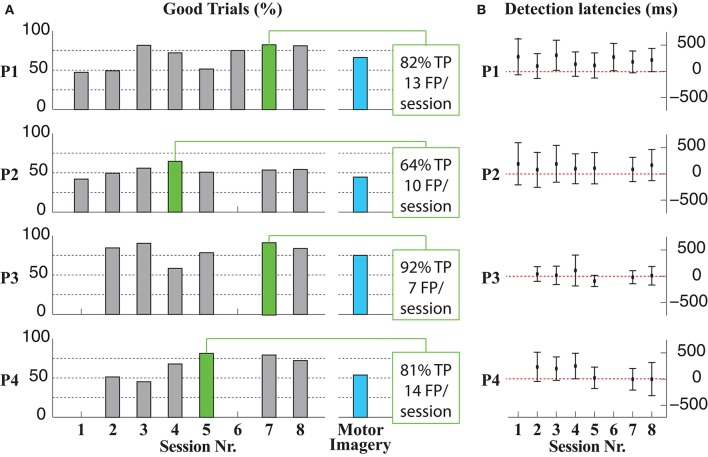
**Summary of the BMI performance along the intervention sessions. (A)** GT results per patient and sessions, including a motor imagery run in the last intervention session with each patient (blue bar). The BMI performance in two sessions (sessions 1 and 6) with patients P3 and P4 could not be estimated due to the loss of the synchronization signal during the recordings. In these cases the interventions could be carried out in equal conditions as in the rest of the sessions **(B)** Detection latencies of the BMI per session and patient.

Average percentages (across sessions) of GT for P1, P2, P3, and P4 were 67.4 ± 15.5, 52.8 ± 6.7, 81.1 ± 12.1, and 66.1 ± 14.8%, respectively (left panel in Figure 4). In the best session for each patient (green bars in the left panel of Figure 4), GT results were 80.9% (P1), 64.4% (P2), 91.7% (P3), and 81.2% (P4). The average TP rate and number of FP per session (considering all stored sessions and patients) were 71.1 ± 19.5% and 8.1 ± 4.9 FP/session, respectively. No statistically significant differences in any measure were found between sessions for all patients [GT: $\chi_{\left(5\right)}^{2}$ = 5.875, *p* = 0.320; TP: $\chi_{\left(5\right)}^{2}$ = 4.000, *p* = 0.549; FP: $\chi_{\left(5\right)}^{2}$ = 5.109, *p* = 0.403].

Results improved along sessions in three cases (see Table 2). TP and GT showed an increasing tendency along sessions for all patient, especially P1, P4, and less markedly in the first sessions of P2. On the contrary, FP showed increasing tendency for P1 and P2 and decreasing for P3 and P4 (see Table 2).

**Table 2:** **Linear least square fitting parameters (*m*, slope; *R*^2^, squared error) of the BMI performance measures along sessions**.

**Pat. Code**	**TP**		**FP**		**GT**		**Latency avg**.	
	***m***	***R*^2^**	***m***	***R*^2^**	***m***	***R*^2^**	***m***	***R*^2^**
P1	5.54	0.460	1.21	0.480	4.50	0.388	−4.99	0.016
P2	1.88	0.397	0.61	0.166	1.06	0.157	−4.13	0.043
P3	0.25	0.001	−0.37	0.029	0.89	0.029	−10.09	0.125
P4	5.64	0.506	−1.66	0.434	4.80	0.564	−38.79	0.636

The average detection latencies (considering all sessions) were 202 ± 266 ms (P1), 130 ± 316 ms (P2), 3 ± 190 ms (P3), and 103 ± 254 ms (P4). No pair of sessions differed statistically in the average detection latency [$\chi_{\left(5\right)}^{2}$ = 7.587, *p* = 0.164].

A tendency toward smaller detection latencies could be observed in all patients (P3, P4, and less in P1, P2) when analysing the evolution along the different sessions (Table 2; right panel, Figure 4).

Unsuccessful results of the BMI-FES intervention were only observed in the sixth session with patient P2 (unreliable estimations of motor intentions were generated in that case). As a result of this unreliable BMI function, this intervention session was interrupted since the patient reported an uncomfortable interaction with the FES.

Figure 4 also includes information on how patients were able to control the BMI-FES interface by performing imaginary instead of actual movements (left panel, blue bars). This condition was tested at the end of the experimentation to ascertain that motor-related activity was robust enough to trigger FES regardless of whether it was accompanied by overt movements. In all cases, GT for motor imagery condition were similar but lower than GT for non-imagined movements.

### Changes in functional scales

Table 3 summarizes the observed changes in the two evaluated functional scales (FMA-UE and SIS) after the intervention period. The FMA-UE score increased in 11.5 points after the intervention, with increases in all patients being observed. All patients showed improvements in the passive range of motion and sensation in FMA-UE scores. In addition, three patients (P1, P2, and P4) showed increases in motor function scores. The SIS score presented an average 10.5 points increase after the intervention. No significant differences in any of the two scales were observed (*p* = 0.114 and *Z* = −1.826 for SIS changes; *p* = 0.068 and *Z* = −1.461 for FMA-UE).

**Table 3:** **Changes in FMA-UE and SIS between pre- and post-intervention assessments**.

**Code**	**FMA-UE**										**SIS**	
	**Motor function**		**Sensation**		**Passive joint motion**		**Joint pain**		**Total**		**Total**	
	***Pre*-**	***Post*-**	***Pre*-**	***Post*-**	***Pre*-**	***Post*-**	***Pre*-**	***Post*-**	***Pre*-**	***Post*-**	***Pre*-**	***Post*-**
P1	12	26	9	10	16	16	24	24	61	76	64	74
P2	31	32	7	8	21	24	24	24	83	88	66	79
P3	24	22	10	12	7	24	24	24	65	82	44	63
P4	28	32	9	10	20	24	24	24	81	90	73	73
Avg. ± *SD*									72 ± 11	84 ± 6	62 ± 12	72 ± 6
**Functional scales**	**Before**								**After**			
FMA-UE	72 ± 11								84 ± 6			
SIS	62 ± 12								72 ± 6			

### Analysis of the kinematics

Table 4 reports joint positions (degrees) when the affected hand reached the glass during the kinematics assessments. On average, after the interventions shoulder flexion was slightly increased (0.7°) and shoulder abduction was reduced (5.45°). Additionally, elbow and thorax flexion were reduced (2.09 and 4.97°, respectively). There were no significant differences after the intervention in any of the joint angular rotations measured (*p* = 0.465 and *Z* = −0.730 for shoulder and elbow flexion changes; *p* = 0.273 and *Z* = −1.095 for shoulder abduction changes, *p* = 0.068 and *Z* = −1.826 for thorax flexion changes).

**Table 4:** **Analysis of reaching movement kinematics before and after the intervention (values represent joints' rotations in degrees)**.

**Joint position (degrees)**	**Before**	**After**
Shoulder flexion	43.8 ± 17.7	44.5 ± 18.4
Shoulder abduction	62.5 ± 42.1	57.1 ± 36.6
Elbow flexion	85.1 ± 16.9	83.0 ± 20.2
Thorax flexion	12.1 ± 4.3	7.1 ± 4.1

### Usability assessment

The perceived comfort and acceptability of the intervention platform proposed here varied across patients. Three patients (P1, P2, P3) “agreed or strongly agreed” with the intervention, while P4 “neither agreed nor disagreed” with it. One patient (P2) reported that the intervention was useful in order to carry out activities of daily living (“strong agreement”), but the other three patients reported “strong disagreement” (P3, P4) or “neither agreement nor disagreement” (P1) in this regard. Regarding the degree of recommendation of the received intervention; all participants declared to “agree or strongly agree.” Finally, two participants (P1 and P4) reported to be in “agreement or strong agreement” with the instrumentation process carried out during the intervention; however, the other two participants indicated to be in “disagreement or strong disagreement.”

## Discussion

This study has tested for the first time the ability of a BMI system to assist upper-limb functional movements of stroke patients based on their pre-movement cortical changes decoded online and on a single-trial basis. Such use of anticipatory EEG activity allows the timely assistance of patients during the motor tasks performed. The study aimed to evaluate the usability of the proposed technology and its potential effects when applied in a prolonged in time intervention. Overall, patients could reliably control the interface by spontaneously performing movements and low average detection latencies (<200 ms) were obtained. Moreover, measured FMA-UE changes were higher than the minimal clinically important difference.

Most BMI interventions involving patients with a stroke have used synchronous paradigms, that is, patients performed movements paced by an external signal, and ERD patterns were used to characterize the movement phases (Ang et al., 2014; Kraus et al., 2015; Bhagat et al., 2016; Irimia et al., 2016). This approach allows a more reliable function of BMIs (FP can be avoided), but it can underestimate the relevance of the temporal coupling between patients' intentions of movements and the perceived afferent feedback. In a series of studies by Mrachacz-Kersting et al., the temporal association between motor intentions and peripheral stimuli proved to be relevant in order to efficiently guide cortical changes related to ankle movements (Mrachacz-Kersting et al., 2012, 2016). While some of these studies involved BMI approaches with healthy subjects, interventions with stroke patients typically used visual cues and fixed (patient-specific, according to BP) stimulation onsets. Such offline BMI-like approach is an excellent solution for practical and robust EEG-based neuromodulation interventions, but it assumes that movements are always performed in identical conditions with respect to the external cues, and hinders the demands on patients for planning movements actively. Being able to have a BMI system asynchronously providing reliable and timely estimations of motor intentions allows higher adaptability to inter-trial changes in movement-related cortical activities and gives rise to using ecological rehabilitation scenarios where patients cannot automatize the task performance according to external guides. In this regard, the present study is an original attempt to demonstrate the suitability of purely asynchronous BMIs for motor neurorehabilitation after a stroke.

Importantly, although a small sample is considered here, results are comparable (and better in some cases) to those obtained in previous similar studies with healthy subjects (Ibáñez et al., 2014a; Xu et al., 2014b; Lin et al., 2016), and are the first demonstration of an online low-latency BMI system tested with patients. The average detection latencies obtained in this study are slightly higher than those obtained in a previous study using the same EEG-based classifier in patients with stroke (112 ± 278 vs. 35.9 ± 352.3 ms). This is probably due to the fact that the previous study was carried out offline while this present study used the BMI online to trigger FES. Since FES was programmed to support patients' movements, it likely had a priming effect on forthcoming movements, i.e., a stimulus arriving when the patient was about to move but with anticipation would in turn anticipate the patients' generation of the intended movement. In any case, detection latencies were in general low enough in order to expect facilitatory effects in the motor cortex (Xu et al., 2014b).

Importantly, the potential impact of motor-related artifacts in the obtained BMI performances, although possible, is estimated to be small. Muscle artifacts, on the one hand, lead to increases of the cortical activity in frequencies within the beta band, which are in the opposed direction to ERD changes learned from the pre-movement EEG signals in the training stage. On the other hand, post-movement low frequency components in the EEG are typically spread along the scalp (which allows spatial filtering techniques to cancel them) and also they typically start with positive changes of the EEG amplitudes (Shibasaki et al., 1980). Furthermore, results obtained with the motor imagery condition tested in the last intervention session are comparable (although smaller) to those obtained with actual movements, which implies that patients with very limited motor capabilities could have the possibility to interact with the proposed BMI-FES platform.

Patients' ERD and BP were marked and started around 0.5–1 s before movements could be observed from gyroscopes. Both patterns showed alterations compared to ERD and BP patterns in healthy subjects in terms of spatial and temporal distributions and in line with previous findings (Serrien et al., 2004; Daly et al., 2006; Fang et al., 2007). ERD patterns tended to show a higher involvement of cortical areas around the vertex (P3 and P4) or of contralesional areas (P2). Regarding the temporal characteristics, BP in patients showed a delayed peak hundreds milliseconds after the actual onset of the movement, in line with previously published studies (Daly et al., 2006). This delayed peak makes it more challenging to generate estimations of intentions to initiate voluntary movements with temporal accuracy. In this study, only the BP part that preceded the movements and finished at *t* = 125 ms was used to model the movement intention class. However, the implications of this decision in the hypothesis that afferent stimulation has to be triggered at the BP peak are not clear, and therefore further research should be carried out to describe the role of post-movement BP parts in patients with stroke. In this study, the validity of the stimuli timings was given not only by their comparison with the actual movements (recorded with gyroscopes) but also by patients' reports indicating that, in most cases, they perceived FES in time with their attempts to perform the reaching task.

Changes in patients as a result of the intervention were observed in terms of changes in the BMI performance across sessions. GT in patients P1, P2, and P4 improved in the first 3–4 sessions, and remained high in P3. In addition, average detection latencies decreased with sessions in P3, P4, and less consistently in P2. These results indicate that patients were able to modulate preparatory cortical activity that released the movements, and therefore reinforces the idea that asynchronous BMI approaches as the one here are suited to reinforce and maximize motor planning in stroke.

Apart from changes in BMI performance, no other neurophysiological changes are described here. Using ERD and BP phenomena simultaneously gives raise to the hypothesis that the intervention might induce changes in both motor planning and execution concurrently, contrasting the existing approaches that singled only one of the former phenomena out. However, despite changes in EEG due to the intervention period were explored, no consistent results were found in terms of ERD, BP, or power in motor cortical rhythms during rest (results not included here). Such changes, if they exist, should be derived from a larger sample of patients, given the high intra-patient variability in EEG information across days (Shenoy et al., 2006). Changes in cortical excitability derived from non-invasive brain stimulation are not reported here either. Such changes have been observed in other neuromodulation interventions with muscles having large cortical representation areas, but not with proximal muscles as the ones here stimulated. From the authors' perspective, the variability obtained in responses to brain stimuli targeting proximal upper-limb muscles requires further investigation in order to use these metrics to validate BMI systems as the one proposed here.

Regarding functional changes, the analysis of reaching kinematics led to small but consistent results. Reaching movements need an adequate range of motion toward thorax extension, shoulder flexion, and elbow extension. The altered kinematic of reaching movements in stroke are typically characterized by compensatory trunk and shoulder movements (Roby-Brami et al., 2003a,b). Taken together, the positions of joints observed after the intervention pointed to an improved reaching movement execution, with higher shoulder flexion (increased 0.7°), lower elbow and thorax flexion (2.09 and 4.97°, respectively). In addition, patients showed a reduction of 5.45° in the compensatory shoulder abduction after the intervention period. In general, a more symmetrical reaching pattern could be observed in most cases. Additionally, FMA-UE scores obtained immediately after the intervention were higher than the minimal clinically important difference (MCID) established for the FMA-UE in chronic stroke patients, which ranged from 4.25 to 7.25 points (Page et al., 2012). All participants showed improvements in the total score in FMA-UE. Improvements were found in passive joint motion scores, which may be related to the repeated training of specific movements with assistance. Additionally, motor function and sensation in FMA-UE scores improved, likely reflecting a favorable effect of repeated motor activity using FES to assist upper-limb reaching (Wang, 2007). These results are in line with previous reports suggesting that combined modulation of voluntary movement, proprioceptive sensory feedback, and electrical stimulation can play a relevant role in improving impaired sensory-motor integration by FES therapy (Hara, 2010). The SIS-16 after the intervention was also increased in the patients (10.5 points). This increment was between the MCID range established for the SIS-16 (9.4–14.1 points; Fulk et al., 2010). These results point to a positive effect of the intervention in the participants' health status.

With respect to the satisfaction assessments, according to the patients two aspects should be improved in order to carry out this intervention in further studies. First, it is necessary to carry out more sessions and more arm movements to achieve a better learning transfer. Second, the instrumentation has to be simplified because this aspect may generate fatigue and discomfort in the participants.

This study was carried out with a small sample of patients. This implies that, despite consistent changes could be observed across patients, no statistically significant changes were observed in the clinical or functional metrics, likely due to the small statistical power. To demonstrate the benefits of the intervention proposed here as compared to (or in conjunction with) more traditional therapies, future studies in line with the present manuscript should involve larger groups of patients, with sample-matched control groups and blind assessments to avoid observer's bias. Moreover, the analysis of the proposed intervention in subacute stroke would also be relevant to test if the functional impact is larger in patients more susceptible to neuromodulation interventions.

## Conclusions

This manuscript represents an approach to BMI-FES interventions for the upper limb in stroke patients, exploiting the predictive properties of EEG signals related to motor processes. Results show a potentially beneficial effect of the BMI-FES intervention in terms of clinical scales and kinematic analysis. In addition, the study demonstrates the suitability of the proposed EEG-based decoding algorithms for their use with patients.

## Author contributions

JI, FM, EM, and JS participated in the design of the experiment, in the data collection process, in the data analysis, and in writing the manuscript. MD and JP designed the experiments, carried out the experimentation, and wrote the manuscript. RC, AC, MC, IA, and JM collected the patients, carried out the experiments, and edited the manuscript.

### Conflict of interest statement

The authors declare that the research was conducted in the absence of any commercial or financial relationships that could be construed as a potential conflict of interest.
