# Corrigendum: Low Latency Estimation of Motor Intentions to Assist Reaching Movements along Multiple Sessions in Chronic Stroke Patients: A Feasibility Study

**DOI:** 10.3389/fnins.2017.00422

**Published:** 2017-07-18

**Authors:** Jaime Ibáñez, Esther Monge-Pereira, Francisco Molina-Rueda, J. Ignacio Serrano, Maria D. del Castillo, Alicia Cuesta-Gómez, María Carratalá-Tejada, Roberto Cano-de-la-Cuerda, Isabel M. Alguacil-Diego, Juan C. Miangolarra-Page, Jose L. Pons

**Affiliations:** ^1^Neural Rehabilitation Group, Spanish National Research Council, Cajal Institute Madrid, Spain; ^2^Sobell Department of Motor Neuroscience and Movement Disorders, Institute of Neurology, University College London London, United Kingdom; ^3^Motion Analysis, Ergonomics, Biomechanics and Motor Control Laboratory, Department of Physical Therapy, Occupational Therapy, Rehabilitation and Physical Medicine, Faculty of Health Sciences, Rey Juan Carlos University Madrid, Spain; ^4^Neural and Cognitive Engineering Group, Centro de Automática y Robótica, Universidad Politécnica de Madrid, Spanish National Research Council Madrid, Spain

**Keywords:** electroencephalography, motor-related cortical potentials, event-related desynchronization, functional electrical stimulation, stroke, neurorehabilitation

In the recently published article, there were incorrect and missing contents in the Acknowledgments section, which should have read as follows.

